# Along with its favorable prognostic role, CLCA2 inhibits growth and metastasis of nasopharyngeal carcinoma cells via inhibition of FAK/ERK signaling

**DOI:** 10.1186/s13046-018-0692-8

**Published:** 2018-02-20

**Authors:** Yuan-Yuan Qiang, Chang-Zhi Li, Rui Sun, Li-Sheng Zheng, Li-Xia Peng, Jun-Ping Yang, Dong-Fang Meng, Yan-Hong Lang, Yan Mei, Ping Xie, Liang Xu, Yun Cao, Wen-Wen Wei, Li Cao, Hao Hu, Qin Yang, Dong-Hua Luo, Ying-Ying Liang, Bi-Jun Huang, Chao-Nan Qian

**Affiliations:** 1 State Key Laboratory of Oncology in South China and Collaborative Innovation Center for Cancer Medicine, Sun Yat-sen University Cancer Center, Guangzhou, Guangdong China; 2 Department of Nasopharyngeal Carcinoma, Sun Yat-Sen University Cancer Center, Guangzhou, China; 3 Department of Radiation Oncology, Longyan First Hospital, Affiliated to Fujian Medical University, Longyan, Fujian China; 4 Department of Pathology, Sun Yat-sen University Cancer Center, Guangzhou, Guangdong, China; 5 Department of Medical Oncology, Sun Yat-sen University Cancer Center, Guangzhou, Guangdong China; 6 Department of Pharmacy, Zhongshan People’s Hospital, Zhongshan, Guangdong China; 7 Department of Traditional Chinese Medicine, the First Affiliated Hospital of Sun Yat-Sen University, Guangzhou, China; 8 Department of Radiation Oncology, Affiliated Cancer Hospital and Institute of Guangzhou Medical University, Guangzhou, China; 9 State Key Laboratory of Oncology in Southern China, Department of Experimental Research, Sun Yat-sen University Cancer Center, 651 Dongfeng East Road, Guangzhou, 510060 China

**Keywords:** Nasopharyngeal carcinoma, CLCA2, Metastasis, Prognostics, FAK/ERK

## Abstract

**Background:**

CLCA2 was reported as a tumor suppressor and disregulated in breast cancer. However, its function in tumor growth and metastasis in NPC has rarely been reported. In this study, we investigated the functional and molecular mechanisms by which CLCA2 influences NPC.

**Methods:**

CLCA2 expression in human NPC cell lines and tissues was examined via real-time PCR (RT-PCR), Western blot and IHC. The biological roles of CLCA2 in proliferative, migration and invasion of NPC cell lines was evaluated in 5-8F, S18, S26 and SUNE-1 cells. Cell viability, migration and invasion were assessed in vitro by MTS, colony formation and transwell assay, respectively. CLCA2 in growth and metastasis of NPC were evaluated in vivo through NPC xenograft tumor growth, lung metastatic mice model and popliteal lymph node (LN) metastasis model.

**Results:**

Overexpression of CLCA2 significantly decreased proliferation, migration and invasion of NPC cells. In contrast, knockdown of CLCA2 elicited the opposite effects. CLCA2 overexpression suppressed xenograft tumor growth and lung, popliteal lymph node (LN) metastasis in vivo. CLCA2 inhibited tumor metastasis through suppressing epithelial-Mesenchymal transition (EMT) and in-activating FAK/ERK1/2 signaling pathway in NPC cells. Immunohistochemical staining of 143 NPC samples revealed that CLCA2 expression was an independent, favorable prognostic factor for overall survival and distant metastasis-free survival of patients. In addition, inhibition of FAK and ERK1/2 reversed CLCA2 silencing-induced tumor cell migration. Furthermore, inhibitors against chloride channels suppressed NPC cellular migration which could have been enhanced by the presence of CLCA2.

**Conclusion:**

CLCA2 suppress NPC proliferation, migration, invasion and epithelial-mesenchymal transition through inhibiting FAK/ERK signaling.

**Electronic supplementary material:**

The online version of this article (10.1186/s13046-018-0692-8) contains supplementary material, which is available to authorized users.

## Background

Nasopharyngeal carcinoma (NPC) is a common malignancy in southern China, Southeast Asia, and North Africa [1–4]. Despite great improvements in radiotherapy and chemoradiotherapy for NPC [5, 6], the outcome of patients with locoregionally advanced NPC is still unsatisfying [7]. This is in part due to the limited efficacy of current therapies against metastasis as well as the emergence of resistance to existing treatments [8, 9]. For these reasons, exploring the molecular mechanisms underlying NPC progression and metastasis to identify new theranostic targets is critical to improving NPC treatment outcomes.

Epithelial-to-mesenchymal transition (EMT) plays a key role in the invasion and metastasis of various epithelial tumors [10–12]. Accompanying the phenotypic and morphologic changes of tumor cells, the expression of mesenchymal markers, e.g., fibronectin, vimentin and N-cadherin will usually increase, while expression of epithelial markers, e.g., E-cadherin and α1-catenin, will decrease [13]. Several studies have shown that inhibiting the EMT process can suppress pulmonary metastasis of tumor cell-bearing nude nice [14], indicating the potential clinical value of targeting EMT in cancer treatment.

Calcium-activated chloride channel (CLCA) proteins belong to a family of plasma membrane Cl^−^ channels that are activated by calcium [15]. Different CLCA proteins have unrelated, sometimes conflicting functions [16]. These functional roles include the regulation of chloride conductance across epithelial cells, epithelial fluid secretion, cell-cell adhesion, apoptosis, cell cycle control, tumorigenesis, and metastasis [17]. CLCA2 is a type I integral transmembrane protein [15]. Recent studies have shown that CLCA2 is a stress-inducible gene and is strongly upregulated by p53 in response to cell detachment, DNA damage, and other stressors [18], rCLCA2 is a novel ultraviolet B(UVB) target gene that may play a role in epidermal differentiation and UV-dependent skin malignancies [19]. CLCA2 is frequently down-regulated in breast cancer and is a candidate tumor suppressor gene for negative regulation of breast cancer cell migration and invasion [20]. One study reported that the roles of CLCA2 in breast cancer include inhibiting proliferation, promoting apoptosis or senescence, and maintaining the cytotoxic response of cancer cells in response to doxorubicin treatment [21].

The roles of CLCA2 in NPC, however, are unknown. In our previously established cellular and animal models of NPC metastasis [22], whole genomic expression profiling revealed that CLCA2 is markedly down-regulated in highly metastatic NPC cells. In the present study, we hypothesized that CLCA2 is a candidate suppressor of NPC development and metastasis. We provide evidence that CLCA2 is negatively correlated with EMT, and reduced expression of CLCA2 in the primary tumor is associated with poorer prognosis in NPC patients. Furthermore, we found that overexpression of CLCA2 inhibits NPC growth and metastasis in vitro and in vivo, and knockdown of CLCA2 by siRNA promotes NPC cell migration and invasion though activation of FAK, which is a key driving force in cancer progression. A small-molecule inhibitor of FAK and ERK1/2 significantly abolished the effects of CLCA2 siRNA in promoting cellular migration.

## Methods

### Cell lines and culture

Four kinds of NPC cell lines SUNE-1, 5-8F, S26, S18 were cultured in DMEM (Gibco, USA) supplemented with 10% Fetal Bovine Serum (FBS, Gibco) with 100 IU/ml streptomycin and 100 IU/ml penicillin, all cells were cultured in a humidified atmosphere of 5% CO2 at 37 °C. (S26 and S18 were isolated from their parental line CNE-2 [19], and 5-8F from its parental line SUNE-1 by limited dilution method [21]).

### Patients tissue samples

A total of 34 primary NPC tissues and 28 non-cancerous nasopharyngeal mucosa (NP) tissues were collected from Department of Nasopharyngeal Carcinoma, Sun Yat-sen University Cancer Center (SYSUCC) and stored at − 80 °C before qRT-PCR analysis. Another cohort of 143 formalin-fixed, paraffin-embedded primary NPC specimens obtained from the Department of Pathology in SYSUCC were used for the immunohistochemical evaluation. All the patients were first diagnosed with NPC both histologically and clinically between February 2006 and December 2010, and the clinical staging system of the International Union Against Cancer was used. Written informed consent was obtained from all patients and the study was approved by the ethics committees of the SYSUCC .

### RNA isolation and real-time quantitative reverse-transcription PCR

Total RNA was isolated from cultured cells using TRIzol reagent (Invitrogen, USA).

Complementary DNA (cDNA) synthesis using the reverse transcription kit (Thermo, K1622) following the manufacturer’s instructions. qRT-PCR analysis was performed using SYBR Green PCR Kit (Novazy, China), and The relative mRNA levels were shown as the value of 2^-△Ct^, (the Ct of β-actin or CLCA2 minus the Ct of the target gene). The sequences of real-time PCR primers were as follows:

β-actin, forward 5′-CACCATTGGCAATGAGCGGTTC-3′and reverse 5′- AGGTCT.

TTGCGGATGTCCACGT -3′; CLCA2, forward 5′-ACAGGCTGGTGACAAAGTG.

GTC-3′ and reverse 5′-ACTGGCAATGCCCACGAAGGTA-3′; β-actin was used as the internal control for measuring the relative level of CLCA2.

### Cell viability assays

Cells were seeded into 96-well plates at the density of 1 × 10^5^ cells/well in 100 μl normal culture medium. Cell growth was determined using MTS (Cell Titer 96 Aqueous One Solution Cell Proliferation Assay solution; sigma) for a week. For everay day, 10 μl MTS reagents were added to 100ul culture medium per well and incubated for 2-4 h at 37 °C. OD490 was determined with a microplate reader. All experiments were performed in triplicates.

### Small interfering RNA transfection

The small interfering RNA was purchased from GenePharma Biological Technology (Shanghai, China) and siRNA sequences targeting human CLCA2 are 5′-CGAAGTTCTGTTACCCATA-3′(si1#) and 5′- GGTCGTTGTATAA.

GTCGAA-3′(si2#). Negative control sequence was 5′-CATTAATGTCGGACAAC.

-3′. Transient transfections of NPC cells were performed as described previously [23] .

### Sphere-formation assay

Cells were cultured in DMEM F/12 medium contained with 20 ng/ml of bFGF and 20 ng/ml of EGF and B27 (Invitrogen) on 6-well low-attachment plates (Corning, Acton, MA, USA) at a density of 1000 cells/well. Under these conditions, the cells grew in suspension as spherical clusters, conditioned medium was changed every 3–4 days. After incubation at 37 °C for 7-10 days, pictures were taken under a microscope and the number of spheres were counted in all wells.

### Lentiviral transduction studies

CLCA2 overexpression plasmid or a vector plasid were purchased from Genecopoeia Co. Ltd. (Guangzhou, China). Lenti-viruses were produced by 293 T cells with CLCA2 using X-treme GENE DNA transfection reagents (Roche). Infectious lentiviruses into 5-8F and S18 cells in 12 h, the medium was exchanged for fresh medium, stably transfected cells were selected with puromycin (2 mg/mL) for 7 days and validated by quantitative RT-PCR and immunoblotting.

### Western blot analysis

The primary antibodies used were CLCA2 (HPA047192,Sigma aldrich, USA), GAPDH(#8884), FAK(#3285), β-actin(#5125), Phospho-p44/42 MAPK(Erk1/2).

(Thr202/Tyr204)(#8544), Phospho-FAK(Tyr925)(#3284), Phospho-FAK(Tyr397)

(#8556) and Phospho-FAK(Tyr576/577)(#3281), E-cadherin(#8834), N-cadherin.

(#4061), snail(#4719), slug(#9585) antibodies were obtained from Cell Signaling Technology (CST, Shanghai, China). The secondary antibodies were HRP-conjugated goat anti-rabbit or anti-mouse antibodies (1:10,000, Vazyme Biotech, china). Proteins were visualized with an enhanced chemiluminescence detection system (biorad, USA).

### Migration and invasion assays

3 × 10^4^ cells of 5-8F, S18 and 5 × 10^4^ cells of S26, SUNE-1 were seeded in a serum-free medium in the top chamber (Corning, USA), and the lower chambers with 700 μl of DMEM with 10% FBS. After cultured for 12-24 h, Cells were stained with methanol and 0.1% crystal violet for 30 min at room temperature. For invasion assays, upper chamber membranes were coated with matrigel (Corning, Life sciences,USA), Three random fields per well were observed, and cells were counted under a microscope. PF-573228 was purchased from selleck. Both experiments were repeated independently three times.

### Wound healing assays

Cells were seeded into 6-well plated and cultured until 90% confluent. After 24 h starvation serum-free medium, using a sterile 200-μL tip to creat artificial wounds in the cell monolayer, and the floating cells were removed by washing with PBS. Respective images were captured at 0 h and 24 h using an inverted microscope. Monitored under a microscope and quantified the width of the scratch between the original and after cell migration.

### Immunohistochemical (IHC) staining

IHC analysis was carried out as previously reported [24]. Rabbit anti-CLCA2 antibody (1:100 dilution, HPA047192, Sigma), Two pathologists scored the intensity of immunohistochemical staining in the tumor cells independently, and the average value from the two pathologists was used as the final score. The percentage of stained cells (0% = 0; 1%–10% = 1; 11%–50% = 2; 51%–80% = 3; 81%–100% = 4), The intensity of the color reaction (no staining = 0; weak staining = 1; moderate staining = 2; strong staining = 3).

### Animal experiments

Female athymic mice between 4 and 6 weeks of age were obtained from Beijing Vital River Laboratory Animal Center. Mice were cared accordance with the principles and procedures outlined of Institutional Animal Care and Use Committee at SYSUCC. The spontaneous LN metastasis experiments has been published previously [25]. Briefly, 2 × 10^5^ cells were injected into the left hind footpad of each mouse. After 4 weeks, the popliteal LNs of the left hind feet were isolated and were homogenized in TRIzol for total RNA extraction. Total RNA (1 μg) was used for cDNA synthesis with 10 ng cDNA used for the following real-time PCR detection of hHPRT with 40 cycles of amplification. The samples with Ct values lower than 30 were identified as metastasis positive. The primers for human HPRT forward: 5′-TTCCTTGGTCAGGCAGTATAATCC-3′; reverse: 5′-AGTCTGG CTTATATCCAACACTTCG-3′; ACTB forward:5′-CAATGAGCTG CGTGTGGC-3′; reverse: 5′-CGTACATGGC TGGGGTGTT-3′.

### Statistical analysis

The SPSS V.14 software package was used to perform statistical data analyses. *P* < 0.05 was considered significant. Data are presented as the mean ± SD. Multivariate analysis was performed using the Cox proportional hazards model. Survival curves were constructed using the Kaplan-Meier method and compared using the log-rank test. All data in our study have been recorded at Sun Yat-sen University Cancer Center for future reference (number RDDB2018000262).

## Results

### Reduced expression of CLCA2 in primary nasopharyngeal carcinoma correlates with distant metastasis and poor prognosis in NPC patients

**Fig. 1:**
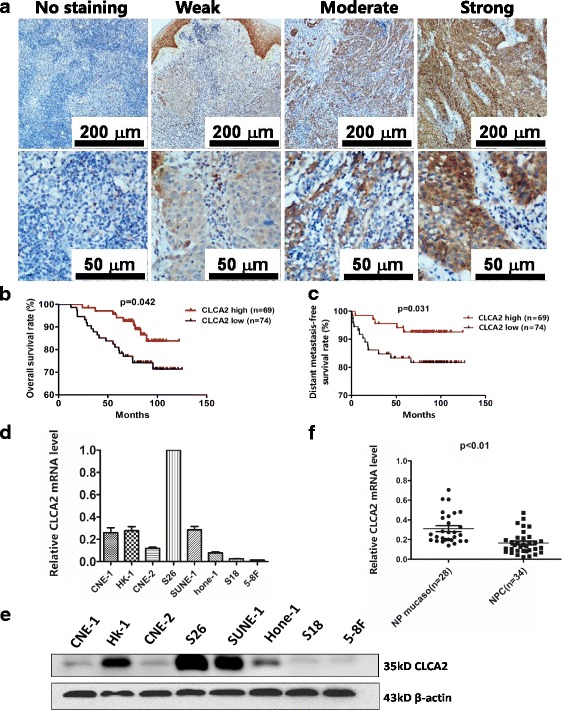
CLCA2 mRNA and protein expression levels in NPC tissues and cell lines. **a** Levels of CLCA2 protein expression in NPC tissues are shown under both low and high magnifications of a light microscope, depressed CLCA2 level correlates with shorter overall survival and distants metastasis-free survival in NPC patients. Scale bars, 200 μm and 50 μm. **b.** The overall survival (OS) rate was significantly higher in the higher CLCA2 group. **c.** The distants metastasis-free survival (DMFS) rate was also significantly higher in the high-CLCA2 group. **d**-**e** CLCA2 was differentially expressed in NPC cell in mRNA and protein levels. **f** Real-time quantitative PCR analysis of CLCA2 mRNA expression levels in NPC (*n* = 34) and normal nasopharyngeal epithelial tissues (*n* = 28),The mRNA levels of CLCA2 in nasopharyngeal carcinoma (NPC) tissues were lower than the noncancerous nasopharyngeal tissues (NP), *P* value, result of unpaired t test

To evaluate the expression level of CLCA2 in NPC tissue, we performed IHC staining in 143 human NPC samples. CLCA2 expression in NPC tissues is shown in (Fig. 1a). The association of CLCA2 expression with clinic pathological features and survival outcomes are summarized in (Tables 1 and 2). Low expression of CLCA2 (cut off score < 3.5) was significantly associated with the WHO histological classification (*p* = 0.03), high death risk (*p* = 0.038), distant metastasis (*p* = 0.04), and disease progression (*p* = 0.038, chi-square test) (Table 1). High CLCA2 expression was associated with significantly longer overall survival (OS) and distant metastasis-free survival (DMFS) than low CLCA2 expression (Fig. 1b and c). Multivariate analysis revealed that high CLCA2 expression was an independent, favorable prognostic indicator for OS and DMFS (*P* = 0.025, *P* = 0.029) in all patients (Table 2). Low levels of CLCA2 may represent a novel prognostic factor in NPC.

**Table 1:** Association between expression of CLCA2 and clinicopathological characteristics in 143 NPC patients

Clinical factor	Cases(*n* = 143)	CLCA2expression		*P* value
		Low	High	
Gendar				
Male	104	53	51	0.759
Female	39	21	18	
Ages				
< 45	76	41	35	0.575
≥ 45	67	33	34	
T stage				
T1-2	35	18	17	0.965
T3-4	108	56	52	
N stage				
N0-1	79	42	37	0.706
N2-3	64	32	32	
Clinical stage				
I-II	12	5	7	0.465
III-IV	131	69	62	
WHO histological classification				
Differentiated non-keratinized (type 2)	3	1	2	**0.03**
Undifferentiated or Poorly Differentiated (type 3)	140	73	67	
Local-regional relapse				
NO	123	63	60	0.754
YES	20	11	9	
Diatant metastasis				
NO	124	60	64	**0.04**
YES	19	14	5	
Progression				
NO	107	50	57	**0.038**
YES	36	24	12	
Death				
NO	114	54	60	**0.038**
YES	29	20	9	

*Abbreviations*: *WHO* World Health Organization,*OS* overall survival, *DMFS* distant metastasis-free survival, *CI* confidence interval, *HR* hazard ratio. Statistical significance (*p* < 0.05) is shown in bold

**Table 2:** Univariate and multivariate analyses of different parameters for overall survival (OS) and distant metastasis-free survival (DMFS) of NPC patients

Variables	Univariate analysis			Multivariate analysis		
	HR	Cl	P	HR	Cl	P
OS						
Gender	1.186	0.540–2.607	0.671	1.148	0.518–2.544	0.734
Age	2.875	1.308–6.319	**0.009**	3.219	1.444–7.176	**0.004**
T stage	1.697	0.647–4.454	0.282	1.218	0.413–3.592	0.721
N stage	1.689	0.812–3.513	0.161	1.653	0.738–3.703	0.222
Clinical stage	23.340	0.095–5718.732	0.262	0.00	0.000	0.975
CLCA2 level	0.457	0.208–1.004	0.051	0.403	0.182–0.891	**0.025**
DMFS						
Gender	0.940	0.339–2.611	0.906	0.879	0.313–2.469	0.807
Age	0.859	0.345–2.136	0.743	.868	0.341–2.210	0.767
T stage	1.280	0.425–3.858	0.661	1.651	0.489–5.569	0.419
N stage	2.860	1.087–7.527	**0.033**	3.069	1.084–8.691	**0.035**
Clinical stage	23.272	0.025–22,096.778	0.368	187,840.553	0.000	
CLCA2 level	0.343	0.123–0.952	**0.040**	0.317	0.113–0.890	**0.0290**

*Abbreviation*: *CI* confidence interval, *HR* hazard ratio. Statistical significance (*p* <0.05) is shown in bold

### CLCA2 is downregulated in high-metastasis NPC cells and NPC tissues

We previously isolated and established cellular clones with different metastatic abilities from parental NPC cell lines [26]. Among these isolates, clone 18 (S18) exhibited the highest metastatic ability, whereas clone 26 (S26) and the parental line CNE-2 had low metastatic abilities. An additional high-metastasis clone, 5-8F, was isolated from a low-metastasis parental NPC cell line, SUNE-1 [22]. CLCA2 mRNA and protein expression levels were initially measured in all tested NPC cell lines. CLCA2 has a very high expression in S26, which mRNA level in CNE-1 was similar to HK-1 and SUNE-1, however, the protein level of CLCA2 in CNE-1 was apparently much lower even compared to Hone-1, the relative expression of CLCA2 was significantly lower in high-metastasis (S18、5-8F) compared to low-metastasis(S26、SUNE-1) cell lines whether in mRNA level or protein level. (Figure 1d and e). We also investigated CLCA2 expression in NPC tissues. Real-time PCR analysis revealed that CLCA2 mRNA was significantly lower in 34 human NPC tissues compared with 28 non-cancerous nasopharyngeal tissues (Fig. 1f). Therefore, we hypothesized that CLCA2 plays a role in suppressing NPC progression.

### Overexpression of CLCA2 inhibits NPC cell growth in vitro and in vivo

**Fig. 2:**
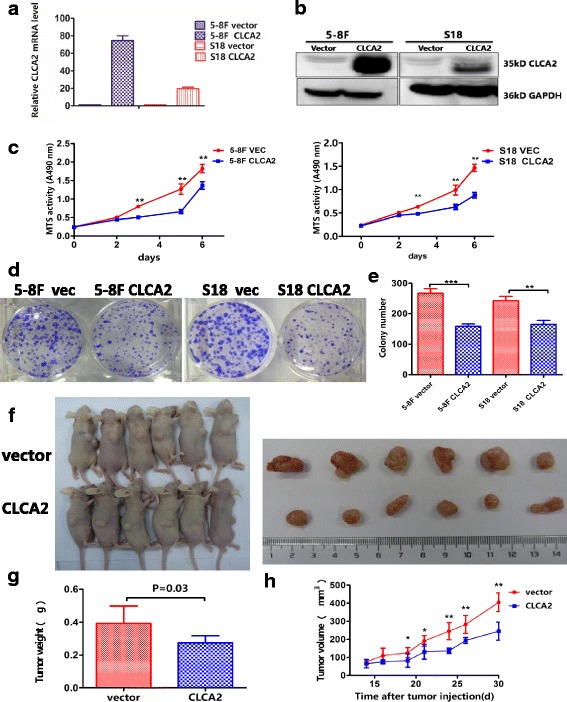
Overexpression of CLCA2 inhibits the growth in NPC cells in vitro and in vivo. **a** Overexpression of CLCA2 in NPC cells were determined by real-time quantitative PCR, normalized to β-actin. **b** Overexpression of CLCA2 in NPC cells were determined by immunoblotting analysis, GAPDH was used as a loading control. **c** Overexpression of CLCA2 inhibits cells proliferation was determined by the MTS assay; **P < 0.01, result of Student t test. **d**-**e** Overexpression of CLCA2 decreased cell colony numbers; representative micrographs and quantification of crystal violet stained cells from 3 independent experiments. **f** Overexpression of CLCA2 in 5-8F cells and the vector control cells were subcutaneously injected into nude mice. **g** The terminal tumor weights are decreased compared with the control group, *P < 0.05, **P < 0.01, result of Student t test. **h** The growth curve indicates 5-8F growth suppression upon CLCA2 overexpression in vivo*,* the results are presented as the mean ± SD, *n* = 6 per group, scale bar, 1 cm

To confirm the role of CLCA2 in NPC development, CLCA2 was stably overexpressed in high-metastasis 5-8F and S18 cell lines. Empty vector-transfected 5-8F and S18 cells were used as controls. The overexpression of CLCA2 in these cells was confirmed by real-time quantitative PCR and western blotting analysis (Fig. 2a and b). In vitro assays revealed that overexpression of CLCA2 effectively inhibited cell proliferation (Fig. 2c) and reduced colony formation ability (Fig. 2d). Meanwhile, we transfected S26 and SUNE-1 cells with siRNA for CLCA2 (si1# and si2#) or negative control siRNA. The siRNA suppression efficiency of CLCA2 protein levels was confirmed by real-time quantitative PCR and immunoblotting (Additional file 1: Figure S1a and b). We observed that CLCA2 suppression significantly increased NPC cell proliferation (Additional file 1: Figure S1c and d) and colony formation ability (Additional file 1: Figure S1e). To examine the effect of CLCA2 on maintaining cancer stem cell characteristics, we used a sphere culture assay and found that overexpression of CLCA2 reduced the number and size of spheres generated from 5-8F and S18 cells (Additional file 1: Figure S1f and g); in addition, protein levels of stem cell markers such as ABCG2, CD44, and β-catenin were decreased (Additional file 1: Figure S1h), confirming the down-regulation roles of CLCA2 in the self-renewal capacity. To further validate our data in vivo, 5-8F cells stably overexpressing CLCA2 sequence (Lenti-CLCA2) or empty lenti-vector (Lenti-vector) cells were subcutaneously injected into the dorsal flank of nude mice, and the size of the tumorigenesis was measured every 3 days. Finally, overexpression of CLCA2 remarkably inhibited tumor growth after tumor formation compared with control group (Fig. 2f). Taken together, These results strongly suggest that CLCA2 acts as a tumor suppressor in the development of NPC.

### CLCA2 influences NPC cell migration, invasion, and metastasis

**Fig. 3:**
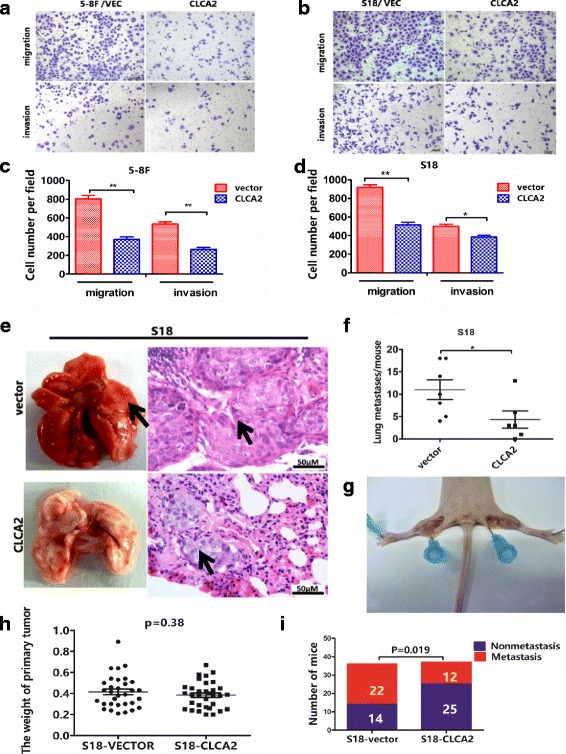
CLCA2 influences NPC cell migration, invasion and overexpressing CLCA2 impairs NPC metastasis in vivo*.* **a**-**b** Overexpression of CLCA2 in NPC cells could inhibit cell migration and cell invasion in 5-8F and S18 cells compared with vector control cells. **c**-**d** Quantification of the effects of CLCA2 overexpression on the migratory abilities and invasion ability in 5-8F and S18 cells as determined by transwell assays, all of the experiments were performed at least three times. Columns, average of three independent experiments; bars, SD. *P < 0.05, **P < 0.01, result of Student t test. **e** Overexpression of CLCA2 decreased in vivo metastatic rate of S18 cells. Histological image of lung metastasis in nude mice after tail vein injection of S18 cells. Left, representative picture of lungs; right, representative H&E staining of lungs; scale bar, 50 μm. **f** The numbers of lung metastases (mean ± SD) were counted and summarized. *P < 0.05. **g** The metastasis rates decreased of overexpression of CLCA2 in S18 cells in vivo, cells (2 × 10^5^) were subcutaneously injected into the left hind footpad of nude mice, and metastasis to the left popliteal LN was measured. **h** The primary tumor weights of left hind footpad are also compared with the control group. *P* > 0.05, Student t test. **i** The proportion of popliteal lymph node metastases was significantly reduced overexpression of CLCA2 in S18 cells (*n* = 36 in vector group, *n* = 37 in CLCA2 group). *P* values were calculated using a chi-square test

**Fig. 4:**
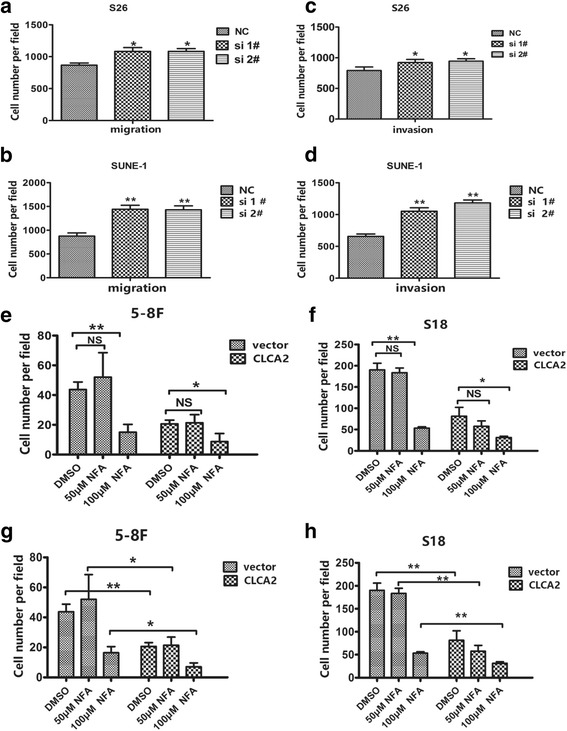
Silencing CLCA2 promotes NPC cell migration and invasion in vitro and CLCA2 enhances the suppressive function chloride channel inhibitor on NPC cell migration. **a**-**b** Quantification of the effects of CLCA2 silencing on the migratory abilities of S26 and SUNE-1 cells as determined by transwell migration assays. Columns, average of three independent experiments; bars, SD. *P < 0.05, **P < 0.01, Student t test. **c**-**d** Quantification of the effects of CLCA2 silencing on the invasion abilities of S26 and SUNE-1 cells as determined by transwell invasion assays. Columns, average of three independent experiments; bars, SD. *P < 0.05, **P < 0.01, Student t test. **e**-**h** Chloride channel inhibitor (NFA) impaired the migration of high-metastasis cells. Cells were treated with 50 μM/L and 100 μM/L NFA or DMSO for 24 h and subjected to migration assays. The quantitative results of migrated cells were calculated by 3 visual fields selected in the transwell assay, means ± SD, *n* = 3, *P < 0.05;**P < 0.01*,* NS*, P >* 0.05, by student t test, scale bar, 100 μm

To examine whether CLCA2 influences NPC cellular mobility, we performed transwell migration, invasion and wound healing assays using two highly metastatic cell lines, 5-8F and S18. Overexpression of CLCA2 dramatically decreased the migration and invasion of 5-8F and S18 cells (Fig. 3a and b); the wound healing assay demonstrated that migration in 5-8F and S18 cells stably overexpressing CLCA2 was much lower than that of cells expressing the vector plasmid (Additional file 2: Figure S2a and b). Meanwhile, we transiently transfected low-metastasis NPC cells, S26 and SUNE-1, with siCLCA2 or control siRNA and performed migration, invasion and wound healing assays. The migration (Fig. 4a and b) and wound healing (Additional file 2: Figure S2c and e) assays revealed that cells transfected with siCLCA2 migrated faster than cells transfected with control siRNA. Silencing CLCA2 promoted NPC cell invasion as determined by the invasion assay (Fig. 4c and d). To validate the metastasis function of CLCA2 in NPC cell in vivo, S18-vector and S18-CLCA2 cells were injected into the tail veins of nude mice. After 6 weeks, mice were euthanized, the number of lung metastatic nodules were significantly fewer in the S18-CLCA2 group than in the control group (Fig. 3e, *P* < 0.05). By hematoxylin and eosin (H&E) staining, we found that the nodules of metastases in the lung tissue of CLCA2-overexpressing mice were significantly fewer and smaller compared to control mice (Fig. 3e and f). To further support our data from NPC lung metastatic model, we established another popliteal lymph node metastasis model. We found that the lymph node metastasis rate was significantly reduced from 61% (22/36) to 35% (12/37) via overexpression of CLCA2 in NPC cells (Fig. 3g–i), but the primary tumor weight in the left hind footpad remained similar (Fig. 3h). Together, these results demonstrate the importance of CLCA2 in the down-regulation of nasopharyngeal carcinoma cellular migration, invasion, and metastasis.

### CLCA2 suppresses NPC cell metastasis by inhibiting the EMT process

EMT is important for the acquisition of metastatic potential in tumors. To explore whether CLCA2 suppresses NPC cell motility through inhibition of EMT, we evaluated the expression of EMT markers by western blotting. Results revealed that overexpression of CLCA2 significantly elevated protein level of the epithelial marker E-cadherin but inhibited protein level of the EMT-inducing transcription factors snail and slug and the β-catenin, N-cadherin. In contrast, silencing CLCA2 suppressed E-cadherin expression but rescued N-cadherin expression (Additional file 2: Figure S2 g). Inconsistency in the observed modulation of different EMT markers was likely due to specificity of the cell lines. Collectively, these data suggest that CLCA2 activation restrains NPC cells from metastasis by preventing EMT.

### CLCA2 enhances suppression of chloride channel inhibitor on cancer cell migration

CLCA is a family of metalloproteases that regulates Ca^2+^-activated Cl^−^ flux in epithelial tissue. In HEK293 cells, CLCA1 promotes membrane expression of an endogenous TMEM16A-dependent Ca^2+^-activated Cl^−^ current [27]. Whole cell patch. -clamp recordings of hCLCA2-transfected HEK-293 cells detected a slightly outwardly rectifying anion conductance that was increased in the presence of the Ca^2+^ ionophore ionomycin and inhibited by DIDS, dithiothreitol, niflumic acid, and tamoxifen [28]. To clarify whether the suppressive role of chloride channel inhibitors could be enhanced by CLCA2, we treated 5-8F and S18 cells with various concentrations of specific chloride channel inhibitor, niflumic acid (NFA), with or without the expression of CLCA2. Interestingly, we found that 100 μM niflumic acid attenuated the migratory ability of 5-8F-CLCA2, S18-CLCA2 and control cells (Fig. 4e and h), which was dramatically enhanced by the presence of CLCA2.

### CLCA2 inhibits nasopharyngeal carcinoma cellular migration and invasion through suppression of FAK/ERK signaling

**Fig. 5:**
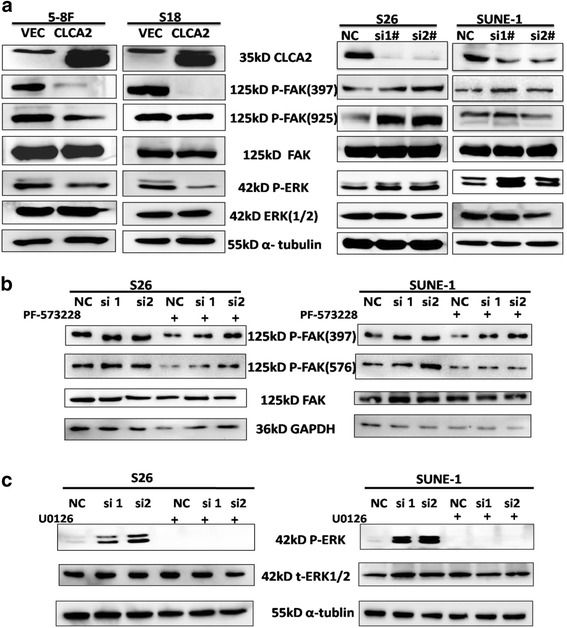
CLCA2 inhibits NPC cellular migration and invasion through suppressing FAK/ERK pathway. **a** Western blotting analysis of P-FAK(397), P-FAK(975), FAK, P-ERK, ERK(1/2) expression levels after overexpression of CLCA2 and silencing of CLCA2. **b**-**c** The expression the FAK/ERK signaling in CLCA2 silencing S26 and SUNE-1 cells after PF-573228 or U0126 treatment was detected by immunoblotting. After treatment with 1 μM PF-573228, 2 μM U0126 or DMSO for 8 h, cell lysates were harvested for western blot detection. Relative gradation corrected by GAPDH is shown below each band

**Fig. 6:**
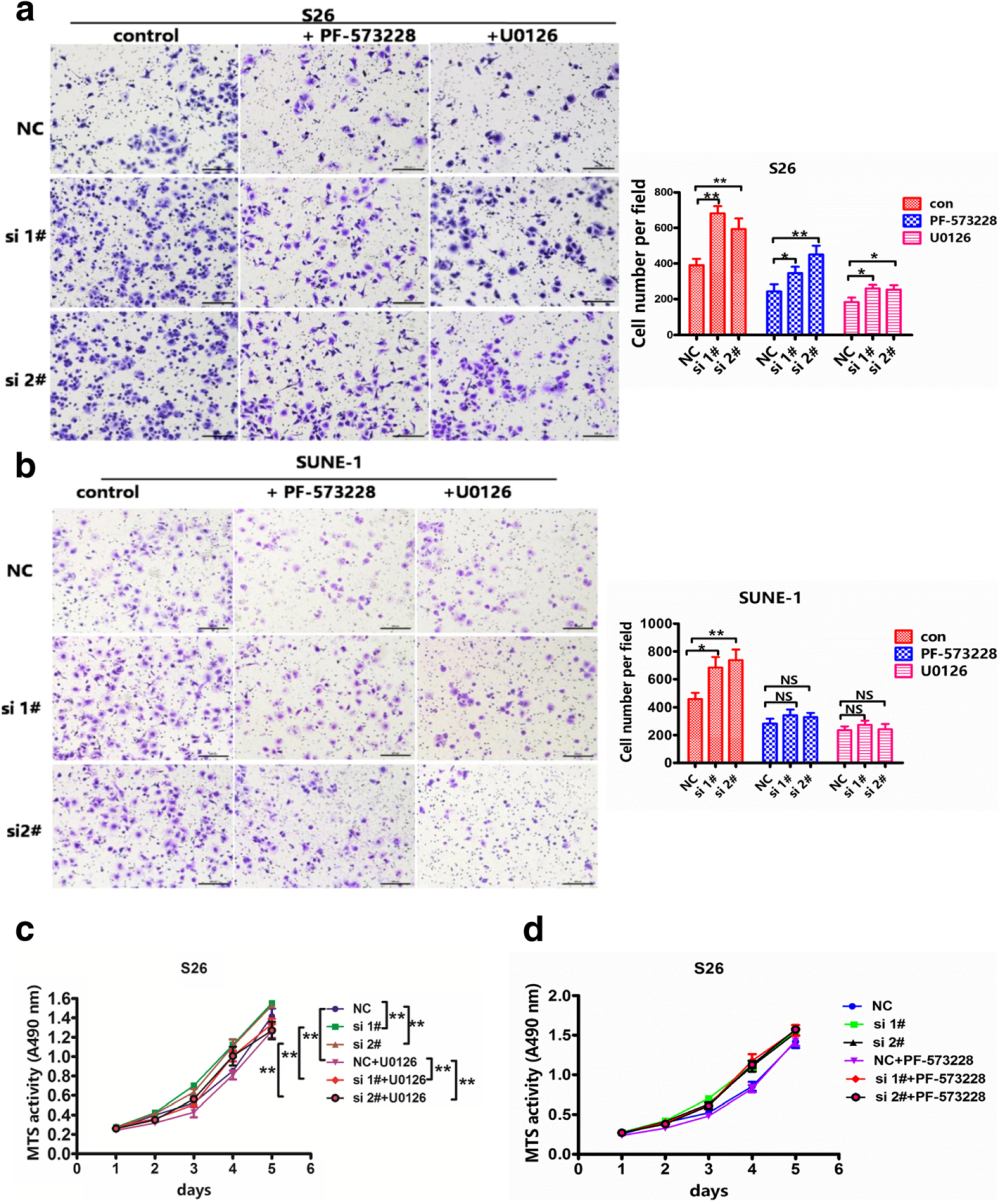
Inhibitors of FAK and ERK1/2 impair the migration and growth of NPC cells induced by CLCA2 silencing. **a**-**b** Cells were treated with 1 μM PF-573228, 2 μM U0126 or DMSO for 24 h, and subjected to migration, representative images and quantification of PF-573228 and U0126 attenuate the effects of CLCA2 silencing on the migratory abilities of S26 and SUNE-1 cells as determined by migration assays. Columns, average of three independent experiments; bars, SD. *P < 0.05, **P < 0.01, Student t test. **c** Cells were treated with 2 μM U0126 or DMSO for 24 h, and subjected to and MTS assays. U0126 attenuate the acceleratedly effects of CLCA2 silencing on the growth abilities of S26 cells as determined by MTS assays. The difference is statistically significant in control and drug groups. **d** Cells were treated with 1 μM PF-573228 or DMSO for 24 h, and subjected to and MTS assays. Treatment with PF-573228 has no influence the effects of CLCA2 silencing on the growth abilities of S26 cells as determined by MTS assays. The difference between control and drug groups has no statistical significance

Focal-adhesion kinase (FAK) is an important mediator of cell proliferation, angiogenesis, cell migration and cell survival. As a result, FAK has been proposed as a new potential therapeutic target in cancer [29]. To examine the role of FAK in CLCA2-inhibited nasopharyngeal carcinoma cellular motility, we used siRNA to knockdown CLCA2 expression in nasopharyngeal carcinoma cells and found that expression of phospho-FAK and downstream phospho-ERK1/2 were enhanced by silencing CLCA2 in S26 and SUNE-1 cells compared with negative controls (Fig. 5a). Conversely, overexpression of CLCA2 decreased expression of phospho-FAK and downstream phospho-ERK1/2 in 5-8F and S18 cells (Fig. 5a). Furthermore, we found that treatment with PF-573228 and U0126 decreased FAK and downstream ERK1/2 phosphorylation in S26-siCLCA2 and SUNE-1-siCLCA2 cells (Fig. 5b and c). These results suggest that CLCA2 inhibits nasopharyngeal carcinoma cell motility via suppression of the FAK/ERK1/2 signaling pathway. To examine whether CLCA2 silencing induces nasopharyngeal carcinoma motility via FAK/ERK activation, we tested the motility of nasopharyngeal carcinoma cells following inhibition of FAK and the downstream ERK1/2 pathway. We found that inhibiting FAK or ERK1/2 impairs migration of S26-siCLCA2 and SUNE-1-siCLCA2 cells (Fig. 6a and b). In addition, the ERK1/2 pathway inhibitor U0126, but not the FAK inhibitor, attenuated cell growth in S26-siCLCA2 cells (Fig. 6c and d). These results illustrate that inhibition of the FAK and ERK1/2 pathway inhibits siCLCA2-stimulated nasopharyngeal carcinoma cells growth and migration.

## Discussion

Recurrent and distant metastases are the main reasons for death in NPC patients. In the present study, we show for the first time that CLCA2 expression is downregulated in NPC cell lines and tissue samples at both the mRNA and protein level. Overexpression of CLCA2 inhibited NPC cell migration and invasion, and overexpression of CLCA2 resulted in fewer pulmonary metastatic tumors in nude mice. High expression of CLCA2 protein in NPC tissues predicts good overall and distant metastasis-free survival of NPC patients. This preliminary study suggests that CLCA2 could be a powerful conservator for anti-metastasis in NPC.

Membrane ion channels are essential for cell fate and appear to have a role in the development of cancer. This was initially demonstrated for potassium channels, cation channels and Cl^−^ channels [30]. Several studies have indicated the importance of chloride and potassium ion current regulation to EMT and invasiveness. The Na^+^-K^+^-2Cl^−^ co-transporter 1 (NKCC1) is an important cell volume regulator that participates in cell migration [31]; ClC-3 chloride channel proteins regulate the cell cycle by up-regulating cyclin D1-CDK4/6 through suppressing p21/p27 expression in nasopharyngeal carcinoma cells [32]; ANO1 is a calcium-activated chloride channel that is up-regulated in various cancers, including gastrointestinal stromal tumor, squamous cell carcinoma of the upper aerodigestive tract and the esophagus (ESCC), and pancreatic cancer [33, 34]. Knockdown of ANO1 in breast cancer cell lines inhibited proliferation, induced apoptosis, and reduced tumor growth in xenografts [33, 35]. In contrast to ANO1, the cystic fibrosis transmembrane conductance regulator (CFTR), mutations of which are responsible for cystic fibrosis, is another chloride channel that acts as a candidate tumor suppressor in breast cancer [20]. Ectopic overexpression of CFTR in breast cancer cell lines downregulates EMT markers and suppresses cell invasion and migration in vitro, as well as metastasis in vivo [36]. The chloride channel accessory (CLCA) protein family has four members in humans (hCLCA1, hCLCA2, hCLCA3, hCLCA4), and all members of this protein family share a high degree of homology in protein size, sequence and predicted structure but differ significantly in tissue distribution and biological function. The CLCA proteins appear to have a role in multiple physiological and pathological processes: chloride conductance across epithelial cells and hence epithelial fluid secretion; cell-cell adhesion, apoptosis, cell cycle control and tumorigenesis and metastasis; and mucous production and cell signaling in respiratory diseases, such as asthma and chronic obstructive pulmonary disease (COPD). Ectopic expression of CLCA family proteins has consistently been shown to produce a novel Cl^−^ current that can be activated by calcium and blocked by typical Cl^−^ channel inhibitors such as TEME16A and CLC-3 [21]. CLCA1 can directly modify TMEM16A activity, increase currents, influence the physiology of multiple tissues and the pathology of multiple diseases including asthma, COPD, cystic fibrosis, and certain cancers [37]. Future studies will determine whether CLCA2 causes TMEM16A expression by interacting with this or other channels. In contrast, some evidence suggests that CLCA2 has only a single, COOH-terminal transmembrane segment followed by a 16 amino acid cytoplasmic tail, a structure incompatible with function as a channel [15]. Similar conclusions have been reached for hCLCA1 and mCLCA3, which are non-integral membrane proteins that cannot be chloride channels, suggesting that they regulate the Cl^−^ current rather than forming an ion-conductive pore [38]. To date, there is no data to unequivocally prove whether CLCA2 forms an anion channel itself or acts as an anion channel regulator. CLCA2 is clustered in the 1p31 region, which is frequently downregulated in breast tumors [39], and loss of hCLCA2 promotes EMT and indicates higher risk of metastasis in breast epithelium [40]. Using the Oncomine database, CLCA2 expression was found to be markedly decreased in primary tumor tissues of the lung, prostate and bladder [41]. In our study, overexpression of CLCA2-negative cell lines significantly reduced tumorigenicity and metastasis, suggesting a tumor suppressor role for CLCA2 in nasopharyngeal carcinoma. Moreover, after treatment with the CLCA inhibitor NFA and CACC_inh_-A01 in 5-8F and S18 cells, cell migration was decreased and this suppressive effect was enhanced by CLCA2, suggesting that chloride channels play a key role in NPC cellular motility.

FAK is an intracellular, highly conserved, non-receptor tyrosine kinase encoded by PTK2 located on human chromosome 8q24.3 [42]. Increased expression of FAK has been reported in tumors of the breast, colon, thyroid, prostate, oral cavity, liver, stomach, skin, head and neck, lung, kidney, pancreas, bone, and ovary [43–45]. FAK is associated with many aspects of metastasis such as adhesion, migration and invasion, but the underlying mechanism of FAK overexpression remains unclear [46]. Focal adhesion kinase (FAK) signaling in cancer cells is regulated by β4 integrin ligation to the mouse endothelial Ca^2+^-activated chloride channel protein mCLCA1 [47]. Targeting FAK pathways that mediate many of these signals has been a major goal in the effort to develop therapeutics. In the present study, overexpression of CLCA2 decreased expression of phospho-FAK and downstream phospho-ERK1/2, blocking signal transmission.

## Conclusions

We first found that CLCA2 was down-regulated in NPC tissues and the low expression of CLCA2 correlates with decreased overall survival and distant matastasis-free survival. CLCA2 suppressed NPC proliferation and metastasis by inhibiting the FAK/ERK signaling pathway, CLCA2 may serve as a potential predictor for distant metastasis and early diagnosis of nasopharyngeal carcinoma.

## Additional files

Additional file 1: FigureS1. Knockdown of CLCA2 promotes the growth in NPC cells in vitro. (PDF 464 kb)

Additional file 2: Figure S2. Overexpressing CLCA2 impairs NPC cell migration and silencing CLCA2 promotes NPC cell migration ability in vitro. (PDF 492 kb)

## Abbreviations

DMFS: Distant metastasis-free survival
EMT: Epithelial-mesenchymal transition
FBS: Fetal bovine serum
H&E: Hematoxylin and eosin
IHC: Immuno-histochemical
NPC: Nasopharyngeal carcinoma
OS: Overall survival
PCR: Polymerase chain reaction
RNAi: RNA interference
