# Supplementary material for: Along with its favorable prognostic role, CLCA2 inhibits growth and metastasis of nasopharyngeal carcinoma cells via inhibition of FAK/ERK signaling
Source: J Exp Clin Cancer Res. 2018 Feb 20;37:34. doi: 10.1186/s13046-018-0692-8 (PMC5819171; doi:10.1186/s13046-018-0692-8)
Supplement: Supplementary file 1 — Knockdown of CLCA2 promotes the growth in NPC cells in vitro. (PDF 464 kb) [file 13046_2018_692_MOESM1_ESM.pdf]

figure S1

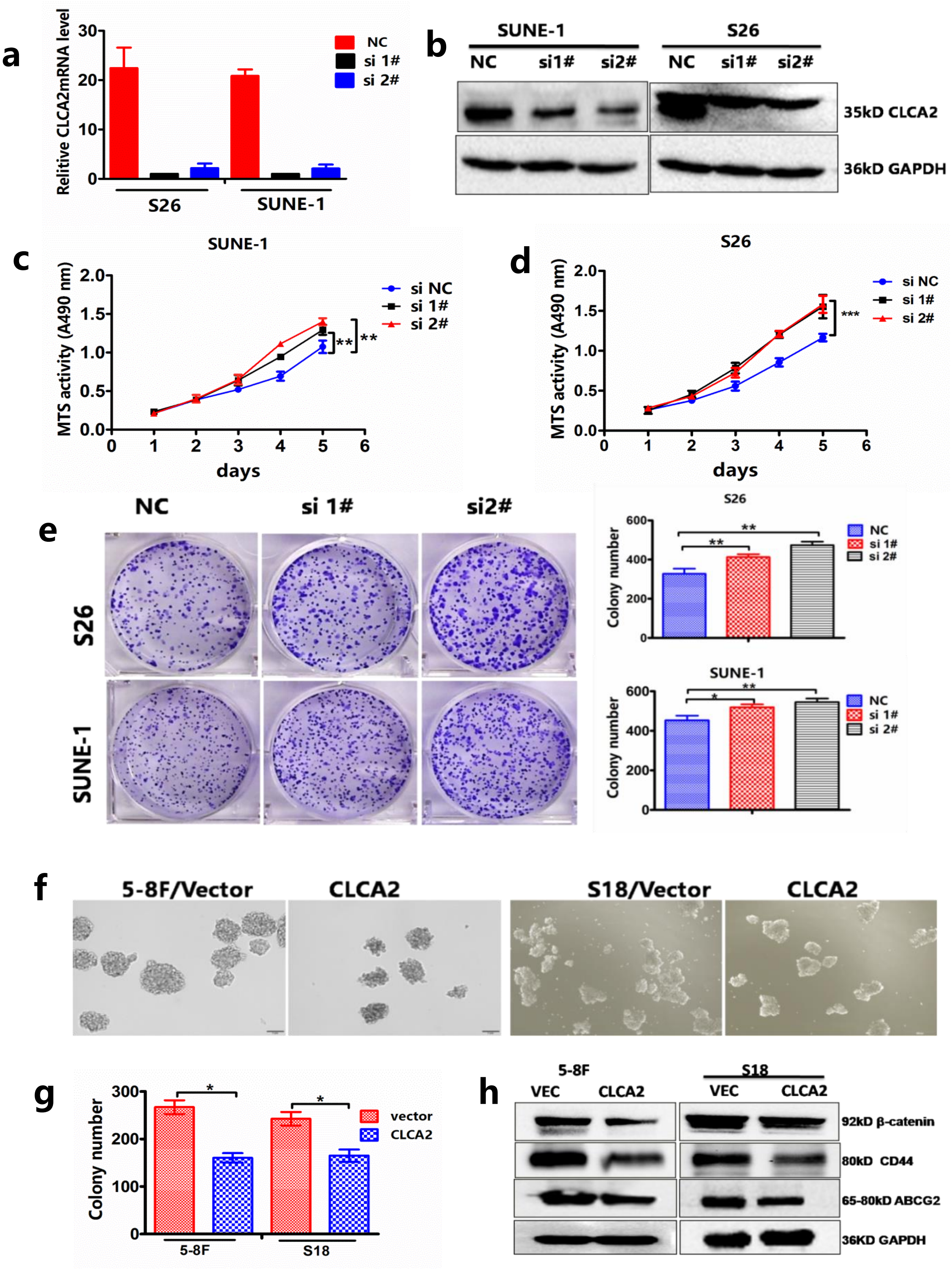

a
Relitive CLCA2mRNA level
30
20
10
0
NC
si 1#
si 2#
S26
SUNE-1
b
SUNE-1
S26
NC
si1#
si2#
35kD CLCA2
36kD GAPDH
c
SUNE-1
MTS activity (A490 nm)
2.0
1.5
1.0
0.5
0.0
si NC
si 1#
si 2#
**
**
days
d
S26
***
e
NC
si 1#
si2#
S26
SUNE-1
Colony number
600
400
200
0
f
5-8F/Vector
CLCA2
S18/Vector
CLCA2
g
Colony number
300
200
100
0
vector
CLCA2
*
5-8F
S18
h
5-8F
S18
VEC
CLCA2
92kD β-catenin
80kD CD44
65-80kD ABCG2
36KD GAPDH

Figure S**1**. Knockdown of CLCA2 promotes the growth in NPC cells in vitro.

a. Silenceing of CLCA2 in NPC cells were determined by real-time quantitative PCR. **b.** Silenceing of CLCA2 in NPC cells were determined by immunoblotting analysis, GAPDH was used as a loading control. **c-d.** Silenceing of CLCA2 promoted the growth in S26 and SUNE-1 cells. Proliferation was determined by the MTS assay; **$P < 0.01$, Student t test. **e.** Silencing of CLCA2 increased the colony formation of NPC cells. Representative micrographs and quantification of crystal violet stained cells from 3 independent experiments. **f-g.** Overexpression of CLCA2 influenced the size and number of spheres. Data represent the average $\pm$ SD, n=3; *$P < 0.05$. **h.** Overexpression of CLCA2 in 5-8F and S18 cells decreased the expression of stem cell-associated genes: β-catenin, CD44 and ABCG2 levels in whole cell lysate samples, GAPDH was used as the loading control.
