# Supplementary material for: Along with its favorable prognostic role, CLCA2 inhibits growth and metastasis of nasopharyngeal carcinoma cells via inhibition of FAK/ERK signaling
Source: J Exp Clin Cancer Res. 2018 Feb 20;37:34. doi: 10.1186/s13046-018-0692-8 (PMC5819171; doi:10.1186/s13046-018-0692-8)
Supplement: Supplementary file 2 — Overexpressing CLCA2 impairs NPC cell migration and silencing CLCA2 promotes NPC cell migration ability in vitro. (PDF 492 kb) [file 13046_2018_692_MOESM2_ESM.pdf]

figure S2

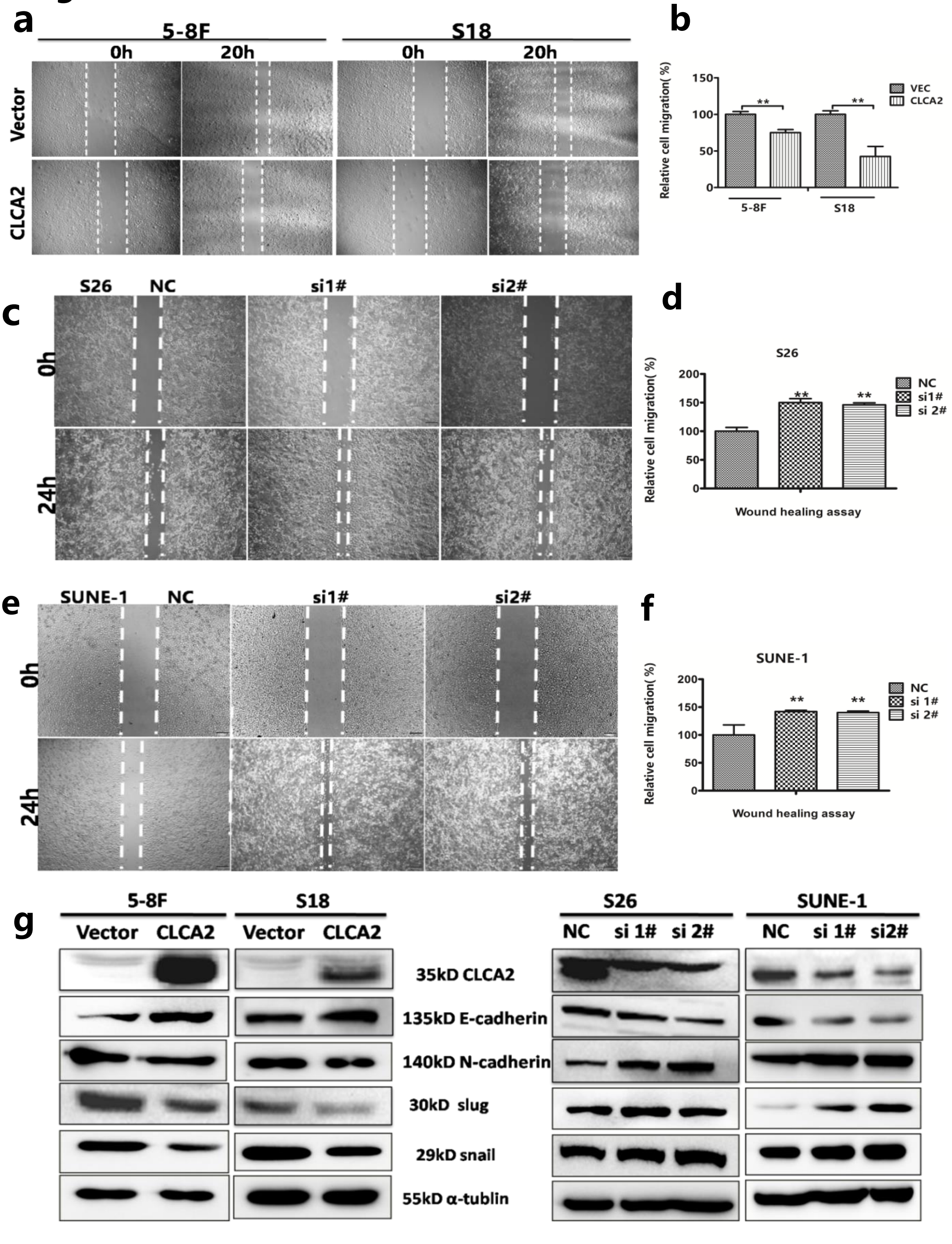

a
5-8F
S18
0h
20h
Vector
CLCA2
b
Relative cell migration(%)
VEC
CLCA2
**
5-8F
S18
c
S26
NC
si1#
si2#
0h
24h
d
S26
Relative cell migration(%)
NC
si1#
si 2#
Wound healing assay
e
SUNE-1
NC
si1#
si2#
0h
24h
f
SUNE-1
Relative cell migration(%)
NC
si 1#
si 2#
Wound healing assay
g
5-8F
S18
Vector
CLCA2
S26
SUNE-1
NC
si 1#
si 2#
si2#
35kD CLCA2
135kD E-cadherin
140kD N-cadherin
30kD slug
29kD snail
55kD α-tublin

Figure S2. Overexpressing CLCA2 impairs NPC cell migration and silencing CLCA2 promotes NPC cell migration ability *in vitro*. **a-b.** Representative images and quantification of the effects of CLCA2 overexpression on the migratory abilities of 5-8F and S18 cells as determined by wound healing assays, all of the experiments were performed at least three times. **c-f.** Representative images and quantification of the effects of CLCA2 silencing on the migratory abilities of S26 and SUNE-1 cells as determined by wound healing assays. Columns, average of three independent experiments; bars, SD. *$P < 0.05$, **$P < 0.01$, Student t test. **g.** Expression of E-cadherin, N-cadherin, snail and slug was measured by western blot in NPC cells in which CLCA2 was overexpressed or silencing, relative gradation corrected by GAPDH is shown below each band.
